# Regional Differences in American Indian/Alaska Native Chronic Respiratory Disease Disparity: Evidence from National Survey Results

**DOI:** 10.3390/ijerph21081070

**Published:** 2024-08-15

**Authors:** Kimberly G. Laffey, Alfreda D. Nelson, Matthew J. Laffey, Quynh Nguyen, Lincoln R. Sheets, Adam G. Schrum

**Affiliations:** 1 Department of Molecular Microbiology and Immunology, School of Medicine, University of Missouri, Columbia, MO 65212, USA; 2 Institute for Data Science and Informatics, University of Missouri, Columbia, MO 65212, USA; 3 Department of Surgery, School of Medicine, University of Missouri, Columbia, MO 65212, USA; 4 Department of Health Management and Informatics, School of Medicine, University of Missouri, Columbia, MO 65212, USA; 5 Department of Biomedical, Biological, and Chemical Engineering, College of Engineering, University of Missouri, Columbia, MO 65212, USA

**Keywords:** American Indian/Alaska Native, disparity, respiratory disease, BRFSS, geographic variation

## Abstract

American Indian/Alaska Native (AI/AN) persons in the US experience a disparity in chronic respiratory diseases compared to white persons. Using Behavioral Risk Factor Surveillance System (BRFSS) data, we previously showed that the AI/AN race/ethnicity variable was not associated with asthma and/or chronic obstructive pulmonary disease (COPD) in a BRFSS-defined subset of 11 states historically recognized as having a relatively high proportion of AI/AN residents. Here, we investigate the contributions of the AI/AN variable and other sociodemographic determinants to disease disparity in the remaining 39 US states and territories. Using BRFSS surveys from 2011 to 2019, we demonstrate that irrespective of race, the yearly adjusted prevalence for asthma and/or COPD was higher in the 39-state region than in the 11-state region. Logistic regression analysis revealed that the AI/AN race/ethnicity variable was positively associated with disease in the 39-state region after adjusting for sociodemographic covariates, unlike in the 11-state region. This shows that the distribution of disease prevalence and disparity for asthma and/or COPD is non-uniform in the US. Although AI/AN populations experience this disease disparity throughout the US, the AI/AN variable was only observed to contribute to this disparity in the 39-state region. It may be important to consider the geographical distribution of respiratory health determinants and factors uniquely impactful for AI/AN disease disparity when formulating disparity elimination policies.

## 1. Introduction

American Indian and Alaska Native (AI/AN) persons in the United States (US) are disproportionately affected by chronic respiratory diseases including asthma and chronic obstructive pulmonary disease (COPD) [1,2,3]. While asthma and COPD have distinct etiologies underscoring the complex interplay of genetic and environmental factors, both diseases can present with exacerbations and require long-term management. Incidents needing medical attention and disease management impact the quality of life for individual patients and impose significant health and economic concerns at the population level [4,5,6]. As such, the disease disparity experienced by the AI/AN population represents an urgent social and public health issue.

Several factors are known to contribute to disease disparity between AI/AN and white populations, including barriers to accessing quality healthcare, adverse socioeconomic indicators, and historical trauma (reviewed in [7]). However, while disease disparity is frequently observed between AI/AN and white populations, the degree of disparity and the potential mechanisms underlying such disparity may not be uniformly distributed across the US, as highlighted by the COVID-19 pandemic [8,9]. Indeed, it has been shown that substantial geographic variation exists in terms of the availability and quality of healthcare providers and state Medicare spending per beneficiary [10,11], which can modify the unequal disease burden borne by other minority populations. Despite the potentially important interplay between regional characteristics and disease disparity, little is known regarding the differential determinants of health in AI/AN populations living in different areas of the US.

To gain a better understanding of the health status of AI/AN persons, the 2017 BRFSS incorporated an oversampling of AI/AN persons in 11 states with relatively high AI/AN populations. Using the oversampled data in 2017, as well as data from other years, we previously showed that the AI/AN variable was not associated with asthma and/or COPD in the 11 states. As approximately half of the US AI/AN population resides in the 11 states [12,13], we tested the hypothesis that the same relationship would hold true for the other half of the population residing in the remaining 39 states and territories.

To do this, we investigated the differential contribution of socioeconomic and behavioral factors to disease disparity between AI/AN and white populations in the US. Using a longstanding historical definition the BRFSS uses to distinguish one broad 11-state region from the rest of the country, we observed a distinction of low- versus high-disease prevalence that occurred irrespective of race. Furthermore, specific to the AI/AN-low geographic region only, the AI/AN variable was independently associated with disease disparity.

## 2. Methods

### 2.1. Data Source

We used data from the BRFSS survey collected in 2011–2019. The BRFSS is an annual random-digit dialed telephone health survey of US residents aged 18 and older [14]. It is conducted by state health departments in collaboration with the Center for Disease Control and Prevention (CDC) in all US states and territories. The analysis of publicly available, de-identified data does not constitute human subject research, as defined in federal regulations, and thus this study did not require Institutional Review Board (IRB) review.

**Figure 1:**
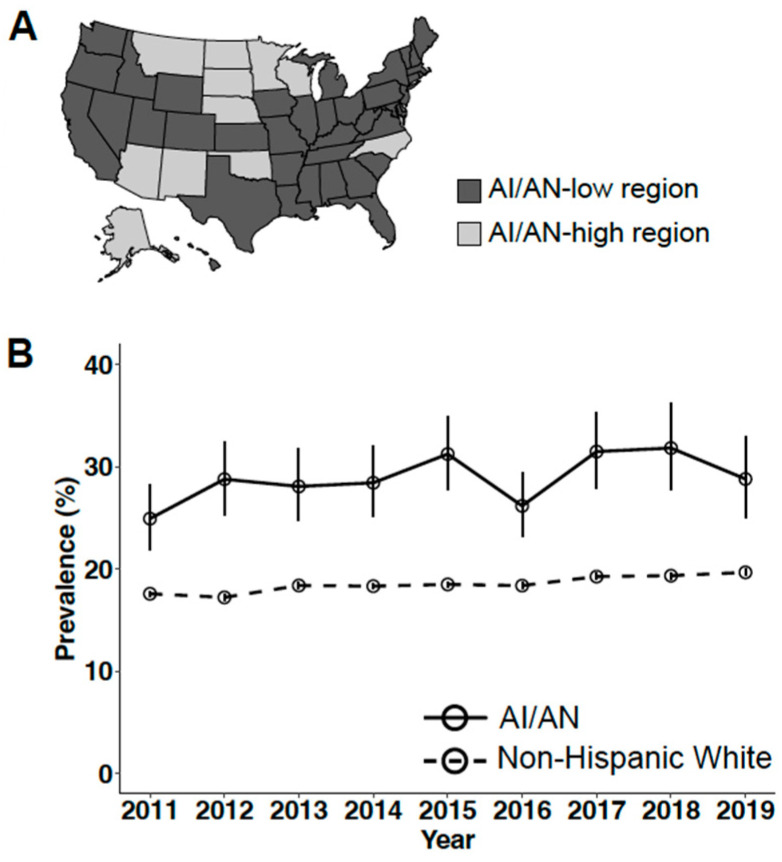
Unequal prevalence and disparity of asthma and/or COPD in the US when dividing the country based on historical AI/AN resident populations. (**A**) An 11-state region (light gray) is historically defined as high in the AI/AN population (AI/AN-high region) and consists of states AK, AZ, MN, MT, NE, NM, NC, ND, OK, SD, and WI. The remaining 39 states (dark gray) comprised a region relatively low in AI/AN population (AI/AN-low region). (**B**) Values of weighted disease prevalence in the AI/AN-low geographic region are plotted with 95% CI. Specific values are presented in Table 1. Comparisons of disease prevalence between the two populations are significant for all years within 2011–2019 using the Rao–Scott χ^2^ test.

In 2017, the AI/AN population was oversampled in 11 states (AK, AZ, MN, MT, NE, NM, NC, ND, OK, SD, and WI) with relatively high AI/AN populations in an effort to gain a better understanding of their health status [15]. We first compared the disease prevalence of the 11-state region with the remaining 39 states and territories. A US map showing the two regions in Figure 1A was generated using the “tigris” package [16]. We then conducted logistic regression to identify variables associated with disease for the 39-state plus territories region.

### 2.2. Measures

The primary outcome variable for this study was chronic respiratory disease status. The exposure variable was race. Covariates included relevant demographic, socioeconomic, and behavioral variables known to contribute to disease disparity [17,18,19,20]. The coding of all variables from BRFSS questions has been described in detail elsewhere [21]. Briefly, chronic respiratory disease status was dichotomized as negative if participants self-reported as having neither asthma nor COPD, or positive if participants self-reported as having either asthma or COPD. The race variable included AI/AN or non-Hispanic White (hereafter called white). Respondents of other races were not included in the analysis. The number of responses analyzed per year averaged 250,759 (235,169–282,472), among which approximately 1% were from AI/AN respondents (0.82–1.2%) (Table S1). Covariates included sex, age, marital status, education level, annual household income, access to healthcare, smoking status, and weight morbidity. Responses with missing values except for the annual household income variable were excluded from analysis. Responses for the annual household income variable contained a substantial proportion of missing values and cannot be assumed as missing at random. These responses were assigned to an “unreported income” category and included it as a level for logistic regression.

## 3. Data Analysis

Bivariable and multivariable logistic regression analyses were performed for each year of BRFSS survey data collected from 2011 to 2019. The Rao–Scott χ^2^ test was used to test for a statistical difference between categorical variables. Two-tailed *p* values < 0.05 are considered statistically significant. To select for significant covariates for the logistic regression models, a stepwise forward modeling process was used. Odds ratios indicate associations when confidence intervals (CIs) exclude 1. To account for the complex sampling design of the BRFSS, data analysis was performed using the “survey” package in R [22] with survey weights specific for each year of the BRFSS data.

## 4. Results

### 4.1. Unequal Prevalence of Chronic Respiratory Disease

According to the American Community Survey (ACS) estimates [12,13], for all years within 2011–2019 included in this study, approximately 50% of the US AI/AN population reside in the 11 states oversampled by the BRFSS in 2017. These 11 states are hereafter called the AI/AN-high region. Having previously observed a higher prevalence of the chronic respiratory diseases asthma and/or COPD in AI/AN compared to white populations in the 11-state region [21], we then sought to analyze disease prevalence disparity between AI/AN and white populations in the remaining 39 US states and territories (AI/AN-low region), where approximately the other half of US AI/AN individuals reside. First, before addressing the race variable, analysis revealed that disease prevalence was not uniform between the 11-state AI/AN-high and 39-state AI/AN-low subregions. The BRFSS-defined 11-state AI/AN-high region showed a relatively lower disease prevalence, while the other 39-state AI/AN-low region showed a relatively higher disease prevalence (Figure 1A). Specifically, for each year analyzed, we found that the AI/AN-high region consistently exhibited a lower disease prevalence for both AI/AN and white populations (min–max prevalence: AI/AN 19.0–23.5%; white 15.5–18.4%, Table 1) compared to their counterparts residing in the AI/AN-low region (min–max prevalence: AI/AN 24.9–31.8%; white 17.2–19.7%, Table 1).

### 4.2. The AI/AN Race/Ethnicity Variable Was Associated with Disease Only in the AI/AN-Low Geographic Region

Comparing the adjusted disease prevalence between AI/AN and white populations, we observed that in the AI/AN-low region, AI/AN residents exhibited a higher prevalence of chronic respiratory disease than white residents (Figure 1B).

We next performed multivariable logistic regression analysis to determine whether the AI/AN racial variable was independently associated with asthma and/or COPD for each survey year. Significant covariates including socioeconomic, demographic, and health-related variables were identified using bivariable analysis (described in Section 2) and were included in the logistic regression models. Surprisingly, unlike results previously reported for the AI/AN-high region [21], the AI/AN race/ethnicity variable was positively associated with disease in the AI/AN-low geographic region, after adjusting for covariates. While we present here detailed results for the most recent survey year included in our analysis (2019; Table 2), the findings were consistent for all survey years (Table S2). In 2019, the AI/AN variable was positively associated with having possessing asthma and/or COPD (OR, 1.33; 95% CI, 1.07–1.66). Other key determinants of disease included low levels of annual income (USD 15,000–<20,000: OR, 1.96; 95% CI, 1.79–2.14; USD 10,000–<15,000: OR, 2.27; 95% CI, 2.06–2.51; <USD 10,000: OR, 2.11; 95% CI, 1.9–2.35), low educational status (less than high school: OR, 1.42; 95% CI, 1.32–1.53), smoking (OR, 1.7; 95% CI, 1.62–1.79), or exhibiting weight morbidity (underweight: OR, 1.31; 95% CI, 1.15–1.50; obese: OR, 1.48; 95% CI, 1.43–1.55). To further evaluate the influence of geographic regions on disease status, logistic regression was performed by including an additional interaction term of region and race on data which included responses from both geographic regions. The interaction term was found to be associated with disease, corroborating the finding that geographical region-specific factors exist to modify disease disparity (Table S3).

## 5. Discussion

In the present study, we have attempted to determine whether the chronic respiratory disease disparity of AI/AN populations might vary with place of residence in the US. Because roughly half of AI/AN persons have historically resided in 11 states in the US, the BRFSS defined that as a region to purposely oversample in 2017, in an effort to increase knowledge of health determinants and wellbeing of the AI/AN population [15]. We used this study’s definition of the 11-state region (AI/AN-high) to compare the analysis with the remaining 39 US states and territories (AI/AN-low), where approximately the other half of AI/AN persons reside. Importantly, we do not interpret any AI/AN cultural or heritage significance to the distinction of these two broad regions defined by the BRFSS study design. Still, we have found that this categorization was sufficient to uncover some salient observations.

First, irrespective of the race variable, the two geographic regions exhibited a difference in disease prevalence. The white population of the AI/AN-low 39-state region reported a higher chronic respiratory disease prevalence than the white population of the AI/AN-high 11-state region, and the same was true of the AI/AN populations comparing the two regions. Second, specific for the AI/AN population residing in the AI/AN-low region only, the AI/AN race/ethnicity variable itself was positively associated with disease. This contrasted with the AI/AN population of the AI/AN-high region where the AI/AN race/ethnicity variable was not associated with disease [21]. Whereas the contribution of the AI/AN variable to disease varied between these two collections of states, socioeconomic determinants remained consistent across both regions of the country (Table S2). In particular, a low annual household income and low levels of education in the dataset were strong determinants of disease, underscoring the interrelatedness of health and socioeconomic status [23,24].

It is important to consider why well-established social and behavioral covariates failed to fully account for the observed disease disparity in the AI/AN population residing in the AI/AN-low region, as this implies the existence of additional factors contributing to disparity. One such factor could be differences in the level of urbanization of the AI/AN communities between the geographic regions. For example, the distribution of AI/AN adults with disabilities and chronic conditions was previously shown to be different from that of all US adults at different levels of urbanization [25]. Further, while our analysis adjusted for the access and utilization of healthcare, it could not capture the quality of healthcare or insurance coverage. The Affordable Care Act (ACA) potentially lowered the barriers for acquiring health insurance for AI/AN persons. Indeed, it was shown that, nationally, the coverage rate of AI/AN persons increased post ACA [26]. However, such increases were not uniform across the country and a coverage disparity between AI/AN and white persons still remained in different regions of the US [27], possibly modifying differences seen in disparity for asthma and COPD. Finally, for AI/AN persons residing in the AI/AN-low region, they might be less likely to obtain services offered by the Indian Health Service (IHS) due to the likely increased physical distance from access. Lack of access to culturally sensitive providers could result in suboptimal care brought about by racial discrimination and cultural differences [28].

What could it mean when the US is split into two large regional groupings and the AI/AN variable contributes to disease disparity in the AI/AN-low region but not the other? To interpret these observations and articulate possible implications, we must first realize the limitations of this study. First, population-level survey data such as the BRFSS dataset must be considered as representing averages across disparate collections of peoples and environments, and no implication about specific tribes and localities can be inferred. Second, we cannot conclude causality from the correlative relationships observed. Third, data such as disease status, race, and income were self-reported and thus might be subject to nonrandom misclassification.

Yet, there is valuable information to be learned. We conclude that there exist additional factors contributing to asthma and COPD unique to the AI/AN-low region as residents exhibit elevated disease rates regardless of race, relative to the AI/AN-high region. We also conclude that disease disparity can involve factors that impact AI/AN persons differentially across geographic regions, a conclusion that could be drawn despite the limitations of the broad categorical division of states in the present study. At this time, the identification of such regionally distributed factors cannot be inferred. We propose that further research with greater focus on culturally informed regions might reveal with specificity how factors contributing to disease disparity may be geographically distributed.

## 6. Conclusions

In this study, we demonstrated that the prevalence for asthma and/or COPD exhibited broad regional differences. The AI/AN race/ethnicity variable was associated with disease only in the subset of states where AI/AN populations are classified as relatively low, historically. Policy deliberations that have as their aim to alleviate disease disparity would benefit from more research on the geographical distribution of factors that contribute to respiratory health. Whereas programs tailored to diminish socioeconomic disparities may effectively lower disease prevalence, more research is needed to identify features unique to certain regions that impact disease disparity experienced by the AI/AN population.

## Figures and Tables

**Table 1:** Yearly weighted prevalence of chronic respiratory disease among AI/AN and white populations in AI/AN-high vs. AI/AN-low regions.

	Weighted Prevalence (95% CI)					
	AI/AN			White		
Year	AI/AN-High	AI/AN-Low	*p* Value	AI/AN-High	AI/AN-Low	*p* Value
2019	20.7 (18.3–23.2)	28.8 (24.9–33.0)	0.001	18.4 (17.8–19.0)	19.7 (19.4–20.0)	4.12 × 10^−4^
2018	23.5 (20.6–26.6)	31.8 (27.7–36.3)	0.002	17.8 (17.2–18.4)	19.3 (19.0–19.7)	2.93 × 10^−5^
2017	20.9 (18.7–23.2)	31.5 (27.8–35.4)	1.55 × 10^−6^	17.5 (16.9–18.1)	19.3 (18.9–19.6)	4.67 × 10^−7^
2016	22.4 (19.9–25.1)	26.2 (23.1–29.5)	NS (0.079)	16.7 (16.2–17.2)	18.4 (18.1–18.7)	1.60 × 10^−7^
2015	19.7 (17.0–22.8)	31.2 (27.8–34.9)	8.48 × 10^−7^	17.3 (16.8–17.9)	18.5 (18.2–18.8)	3.93 × 10^−4^
2014	19.9 (17.9–22.2)	28.4 (25.1–32.0)	2.41 × 10^−5^	16.7 (16.2–17.2)	18.3 (18.0–18.6)	3.07 × 10^−8^
2013	22.5 (19.6–25.7)	28.1 (24.7–31.7)	0.019	17.2 (16.6–17.8)	18.4 (18.1–18.7)	4.57 × 10^−4^
2012	19.0 (16.8–21.3)	28.8 (25.3–32.5)	2.47 × 10^−6^	15.5 (15.1–16.0)	17.2 (16.9–17.5)	8.14 × 10^−9^
2011	19.9 (17.4–22.7)	24.9 (21.8–28.3)	0.019	16.3 (15.7–16.8)	17.6 (17.3–17.8)	8.43 × 10^−5^

*p* Value: *p* values were obtained using the Rao–Scott χ^2^ test. NS: not significant.

**Table 2:** Association between race and chronic respiratory disease in AI/AN-low region for 2011–2019.

	OR (95% CI)	
Year	White	AI/AN
2019	1 (Reference)	1.33 (1.07–1.66)
2018	1 (Reference)	1.53 (1.26–1.87)
2017	1 (Reference)	1.48 (1.24–1.77)
2016	1 (Reference)	1.28 (1.08–1.51)
2015	1 (Reference)	1.58 (1.33–1.88)
2014	1 (Reference)	1.36 (1.13–1.64)
2013	1 (Reference)	1.34 (1.13–1.58)
2012	1 (Reference)	1.50 (1.25–1.81)
2011	1 (Reference)	1.26 (1.06–1.51)

Adjusted for age, sex, income, marital status, education, access to care, smoking status, and body weight morbidity.

## Data Availability

Annual BRFSS data are publicly available at https://www.cdc.gov/brfss/annual_data/annual_data.htm (accessed on 9 February 2022).

## Supplementary Materials

The following supporting information can be downloaded at https://www.mdpi.com/article/10.3390/ijerph21081070/s1, Table S1: Number of responses included in yearly analyses; Table S2: Determinants of chronic respiratory disease for 2011–2019; Table S3: Alternative logistic regression model for determinants of chronic respiratory disease.
